# Surgical outcomes for spinal arachnoid cysts and webs: a case series

**DOI:** 10.1007/s00701-025-06665-7

**Published:** 2025-09-19

**Authors:** Lara Imwinkelried, Danial Nasiri, Ralph T. Schär, Johannes Goldberg, Levin Häni, Andreas Raabe, Christopher Marvin Jesse

**Affiliations:** 1 Department of Neurosurgery, Inselspital, Bern University Hospital, and University of Bern, Bern, Switzerland; 2 Present Address: Department of Neurosurgery, University Hospital Basel, Basel, Switzerland; 3 Clinic for Neurosurgery, Neurochirurgie Am Jahnplatz, Bielefeld, Germany

**Keywords:** Spinal arachnoid cysts, Spinal arachnoid webs, Surgical outcomes, Intradural spinal arachnoidopathies

## Abstract

**Background:**

Spinal arachnoid cysts (SAC) and spinal arachnoid webs (SAW) are intradural pathologies, often presenting with pain and/or myelopathic symptoms. Reports on postoperative outcomes for SAC and SAW are scarce. This study aims to assess the surgical outcomes of SAC and SAW.

**Methods:**

Patients who underwent surgical treatment for SAC or SAW at our institution between 2011 and 2023 were retrospectively reviewed. Demographic data, radiological findings, surgical management, and clinical outcomes were evaluated using the modified McCormick (mMC) scale. Outcomes were categorized as "favorable" (mMC I-II) or "unfavorable" (mMC III-V).

**Results:**

A total of 35 patients (29 SAC, 6 SAW) were analyzed. Mean age was 50.9 (SD ± 9.8) years. Males comprised 70.3% of cases (SAC: 64.5%, SAW: 100%). Most common symptoms were local or radiating pain (SAC 77.4%; SAW 100%), gait-disturbance (SAC 45.2%; SAW 16.7%), sensory-deficits (SAC 32.3%; SAW 66.7%) and impaired motor function (SAC 20%; SAW 16.7%). Median preoperative mMC was 2 in both groups (SAC IQR: 1; SAW IQR: 1). Median postoperative mMC was 1 in both groups (SAC CI 1.07–1.34; SAW CI 0.74–1.60). Favorable outcome (postoperative mMC I-II) was achieved in 26 SAC patients (90%) and 5 SAW patients (83%) respectively. Surgical complications occurred in 14.7%, all in the SAC group, without lasting deficits.

**Conclusion:**

Surgical treatment for SAC and SAW appears to have an overall favorable outcome, though larger cohort analyses are needed. Surgery should be considered in symptomatic patients.

## Introduction

Spinal arachnoid cysts (SAC) and spinal arachnoid webs (SAW) are both uncommon, intradural pathologies. They may cause similar clinical symptoms, including pain, sensorymotor deficits and myelopathy. MRI may not reliably allow to distinguish between the two entities. Both may present with a so called “scalpel sign” a dorsal, focal indentation and may have an associated syrinx [15]. In some cases, only intraoperative exploration allows definite differentiation of the two.

SAC can form as idiopathic primary lesions, or as secondary lesions following subarachnoid hemorrhage, infection, traumatic spine injury, lumbar puncture, intradural surgery or intradural injection leading to an inflammatory arachnoidopathy [3, 11]. The most common hypothesis of formation is an origin from the septum posticum, which is a fenestrated membrane spanning from the pia to the dorsal aspect of the arachnoid in the spinal canal [14, 16]. They are most commonly located in the dorsal thoracic spine [5, 7, 11, 17, 20], while secondary SAC are more likely to present lateral or anterior to the spinal cord [11]. About 20—50% are associated with a syrinx [11, 15, 17, 20]. Symptomatic presentation often consists of myelopathic symptoms with motor deficits, sensory deficits, spasticity and sometimes sphincter dysfunction [3, 5]. Patients with secondary SAC are reported to present with more severe neurological deficits [11]. MRI is the diagnostic modality of choice. While the scalpel sign is a pathognomonic feature of dorsal thoracic arachnoid webs, it may also be present in SACs [15]. SACs will usually present as non-contrast-enhancing and CSF-isointense on MRI. CT-myelography can offer additional diagnostic value, when MRI fails to demonstrate the cyst. Possible surgical treatment modalities are cyst resection, cyst fenestration or placement of a shunt [3]. Outcome measurements throughout studies vary greatly [3]. Reported recurrence rates span from 0%−17% [7, 11, 16, 20] with a higher recurrence rate reported for secondary SAC’s [5], especially post-hemorrhagic [17] with a mere 15% progression-free survival after 10 years [11].

SAW are a rare focal thickening of the arachnoid, causing spinal cord compression. 50% are reported having a history of traumatic spine injury [19]. Another hypothesis for a mechanism of formation is a remnant of a ruptured arachnoid cyst [2, 4]. They are most often located in the dorsal, thoracic spine [19] and often associated with a syrinx (41%—83%) [6, 19]. Common symptoms are pain, motor weakness, sensory loss and gait disturbance. Severe cases may present with pyramidal signs [19]. MRI is the diagnostic modality of choice, where the SAW can be directly visualized and show the so called “scalpel sign” [15]. Treatment by surgical resection is recommended for symptomatic patients [4].

There are a few studies reporting the outcome of these entities. Our aim was to present our cohort of these two seemingly similar entities to shed more light on the outcome of surgical treatment.

## Methods

We conducted a retrospective, single-center surgical case series. All surgical procedures performed at our institution between January 2011 and December 2023 were reviewed to identify patients who underwent surgery for either a spinal arachnoid cyst (SAC) or a spinal arachnoid web (SAW). Relevant clinical and operative data were extracted from electronic medical records. Only patients with a definitive intraoperative diagnosis of either SAC or SAW were included in the final analysis. Patients whose imaging findings were more consistent with a diffuse arachnoidopathy, for example in cases with cysts extending across multiple levels, demonstrating several cyst compartments, or demonstrating circumferential distribution, were excluded. One of these findings alone did not lead to exclusion. Additionally, individuals with a history of prior surgical intervention at the affected spinal level were not included. Refusal to provide general consent led to exclusion of patients.

### Diagnosis, surgical technique and follow-up

Diagnosis was primarily established based on magnetic resonance imaging (MRI). In cases where MRI findings were inconclusive, additional evaluation with computed tomography (CT) myelography was performed. Surgical indication was determined by the presence of clinical symptoms correlating with radiological findings. The specific surgical technique was selected at the discretion of the operating surgeon, guided by both preoperative imaging and intraoperative findings. All procedures were performed via a posterior approach, utilizing either single- or multilevel hemilaminectomies or laminectomies, depending on the anatomical location and extent of the lesion. Following dural opening, intradural exploration was carried out under the operating microscope. SACs were treated by either fenestration or resection, while all SAWs were microsurgically dissected and released. Intraoperative ultrasound was routinely employed to assess spinal cord decompression and restoration of cerebrospinal fluid (CSF) flow. Postoperative follow-up was conducted in the outpatient clinic approximately two months after surgery and included clinical evaluation and follow-up MRI.

### Primary and secondary endpoints

The primary outcome measure was the modified McCormick Scale (mMC) at the time of last follow-up. The mMC is a validated grading system commonly used to assess functional status in patients with spinal cord pathology, including intramedullary tumors and compressive lesions [12]. It evaluates neurological function and the degree of independence in daily activities, with scores ranging from Grade I (normal function or mild deficit without functional limitation) to Grade V (paraplegia or quadriplegia). For the purposes of this study, grades of mMC I-II were classified as a"favorable"outcome, while grades III through V were considered indicative of an"unfavorable"outcome (Table 1).

**Table 1:** The modified McCormick scale [12]

Grade	Modified McCormick Scale
I	Intact neurologically, normal ambulation, minimal dysesthesia
II	Mild motor or sensory deficit, functional independence
III	Moderate deficit, limitation of function, independent with external aid
IV	Severe motor or sensory deficit, limited function, dependent
V	Paraplegia or quadriplegia, even with flickering movement

For secondary outcomes we assessed comprised pre- and postoperative symptoms, surgical technique, disease etiology, imaging modality (MRI and/or CT myelography), and specific radiological features—including intramedullary hyperintense signal on T2 MRI, presence of the scalpel sign, number of involved vertebral levels, lesion location relative to the spinal cord and presence of a syrinx. The British Medical Research Council (BMRC) scale was used to assess motor strength, where 0/5 indicates complete paralysis and 5/5 indicates normal strength.

### Statistical analysis

Descriptive statistics included calculation of the mean, median, standard deviation (SD) and interquartile range (IQR). Statistical analysis was performed with SPSS version 29.0.2.0 (Released 2024, Armonk, NY: IBM Corp). The McNemar Test was performed to analyze change in pre- and postoperative dichotomized mMC grades over all patients in the SAC and SAW Group. A p-value < 0.05 was considered significant.

## Results

### Patient demographics

Between 2011 and 2023, we identified 37 patients who underwent surgical treatment for either a SAC (31) or a SAW (6) at our institution. Two SAC patients were lost to follow-up and excluded from postoperative analysis. The mean patient age was 50.9 years (SD ± 9.8; range 4.5–80). Among SAC patients, 64.5% were male (male-to-female ratio 1.8:1), while all SAW patients were male. Most SACs were primary lesions (67.7%) and predominantly located in the thoracic spine (74.2%). All SAWs were primary and situated dorsal to the spinal cord (Table 2).

**Table 2:** Patient demographics

		Spinal arachnoid cysts (*N* = 31)		Spinal arachnoid webs (*N* = 6)	
		Mean (± SD) / Median (IQR)	*N*(%)	Mean (± SD) / Median (IQR)	N(%)
Age (years)		51.5 (± 9.8)		48.3 (± 16.7)	
Sex	Female		11(35.5%)		0(0.0%)
	Male		20(64.5%)		6(100%)
Primary	Yes		23(74.2%)		6(100%)
Etiology if Secondary	Posttraumatic		3(9.7%)		0(0.0%)
	Postsurgical		4(12.9%)		0(0.0%)
	Posthemorrhagic		1(3.2%)		0(0.0%)
Level	Cervical		2(6.5%)		0(0.0%)
	T1-6		13(41.9%)		4(66.7%)
	T7-12		10(32.3%)		2(33.3%)
	Lumbar		2(6.5%)		0(0.0%)
	Sacral		1(3.2%)		0(0.0%)
	Multiple segments		3(9.7%)		0(0.0%)
Expansion (number of VB)		3(IQR 2.0)		1(IQR 1.0)	
Location	Anterior		2(6.5%)		0(0.0%)
	Posterior		27(87.1%)		6(100%)
	Anterior & posterior		2(6.5%)		0(0.0%)

*N* number, *SD* standard deviation, *T* thoracic, *VB* vertebral body, *IQR* interquartile range

### Preoperative symptoms

In the SAC group, 24 patients (77.4%) presented with local or radiating pain, with a median Numeric Pain Rating Scale (NRS) score of 3 (IQR 6.0). Neurological symptoms included gait disturbance in 14 patients (45.2%), sensory deficits in 10 patients (32.3%), and bladder dysfunction in 9 patients (29.0%). Motor deficits (BMRC grade 4/5 or 3/5 at worst) were observed in 20% of patients (Table 3). The median preoperative mMC was 2 (IQR 1.0). Thirteen patients (41.9%) had a grade of I or II, 4 (12.9%) had a grade of III, 1 patient (3.2%) scored IV, and none scored V.

**Table 3:** Preoperative symptoms in spinal arachnoid cysts and webs

		Spinal arachnoid cysts (*N* = 31)		Spinal arachnoid webs (*N* = 6)	
		Median (IQR)	*N*(%)	Median (IQR)	*N*(%)
Symptom duration	months	29.4(± 93,0)		12.5(± 10.3)	
Preoperative mMC		2(IQR 1.0)		2(IQR 1.0)	
Pain on NRS		3(IQR 6.0)		4(IQR 4.0)	
Worst motor score	1		0(0)		0(0)
	2		0(0)		1(16.7)
	3		2(6.7)		0(0)
	4		4(13.3)		0(0)
	5		24(80)		5(83.3)
Sensory Deficit	No		21(67.7)		2(33.3)
	Yes		10(32.3)		3(66.7)
Gait Disturbance	No		17(54.8)		5(83.3)
	Yes		14(45.2)		1(16.7)
Bladder Dysfunction	No		22(71)		5(83.3)
	Yes		9(29)		1(16.7)
Sphincter Dysfunction	No		27(87.1)		5(83.39)
	Yes		4(12.9)		1(16.7)
Sexual Dysfunction	No		26(89.7)		6(100)
	Yes		3(10.3)		0(0)

*N* number, *IQR* interquartile range, *mMC* modified McCormick Score, *NRS* numeric pain rating scale

In the SAW group, all six patients (100%) presented with local or radiating pain, with a median NRS of 4 (IQR 4.0). One patient demonstrated significantly more severe neurological deficits, including motor weakness (BMRC M2), gait impairment, bladder, and sphincter dysfunction. The median preoperative mMC was 2 (IQR 1.0); 2 patients (33.3%) scored I, 3 patients (50%) scored II, and 1 patient (16.7%) scored V (Table 3).

### Radiological findings

**Fig. 1:**
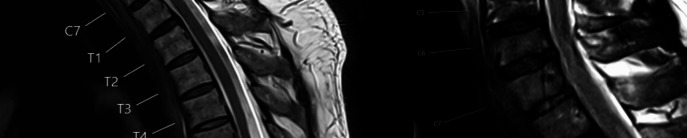
**A** Preoperative T2 sagittal MRI of 58-year-old male, depicting a SAC on levels T5-7. He presented with back pain and hyperreflexia and was surgically treated with a complete resection. Postoperatively the pain subsided, and he showed no new neurological deficits. **B** Preoperative T2 sagittal MRI of a 54-year-old male, depicting a SAW on level T3 with a proximal syrinx. He presented with radiating pain in his right arm, paresthesia of the right arm and was treated with a full resection. Postoperatively the pain subsided. The paresthesia was reduced but still present upon discharge and completely resolved upon last follow-up

All patients had a diagnostic MRI. 6 patients in the SAC and 4 patients in the SAW group underwent additional myelography due to uncertainty of MRI findings. A scalpel sign was found in 13 (41.9%) of SAC patients and in 4 (66.7%) of SAW patients. Two (6.5%) of all SAC patients had a syrinx, compared to 2 (33.3%) of all SAW patients. 10 (32.3%) patients in the SAC group showed an intramedullary hyperintense signal on T2 MRI compared to 2 (6.5%) patients in the SAW group. In both groups 50% of the syrinxes were on the same level as the causing pathology and 50% were distal to the lesion (Fig. 1).

### Surgical approach and complications

All patients underwent posterior surgical approaches via either hemilaminectomy or laminectomy. In the SAC group, 25 patients (83.3%) received a hemilaminectomy and 5 (16.7%) a laminectomy. In the SAW group, hemilaminectomy was performed in 4 patients (66.7%) and laminectomy in 2 (33.3%). SACs were treated with either complete resection (12 cases, 40.0%) or fenestration (18 cases, 60.0%). All SAWs were managed by resection. Duraplasty was performed in 6 SAC cases (20%) and in none of the SAW cases. Mean operative time was 170 (SD ± 81) minutes for SAC and 141 (SD ± 41) minutes for SAW.

The overall complication rate was 14.7% (Table 4), with all complications occurring in the SAC group. Two patients experienced two complications each. All complications resolved fully by last follow-up, with no lasting deficits. Median hospitalization duration was 6 days (IQR 3.0) for SAC patients and 5.5 days (IQR 10.25) for SAW patients. A total of 34 patients (91.9%) were discharged home, while 3 required transfer to a rehabilitation facility.

**Table 4:** Complications

	Spinal arachnoid cysts (*N* = 29)		Spinal arachnoid webs (*N* = 6)
Surgical complications	Epidural hematoma requiring surgical revision Wound healing disorder requiring revision CSF leak requiring revision Positioning damage (skin abrasion)	2 1 1 1	-
Non-surgical complications	Urinary tract infection Postoperative Delirium	1 1	-
Overall Complications		7 in 5 patients	0

*CSF* cerebro spinal fluid

## Postoperative symptoms

The median follow-up duration was 3.9 (IQR 11.7) months for the SAC group, 2.4 (IQR 1.8) months for the SAW group, and 3.6 (IQR 9.5) months overall. All patients underwent follow-up MRI.

In the SAC group, 11 patients (37.9%) showed improvement of at least one grade on the mMC at last follow-up, 16 (55.2%) remained unchanged, and 2 (6.9%) experienced deterioration by one grade.

Of the two SAC patients with worsening mMC grades, one developed new lower limb paresis (BMRC M4) following fenestration of a T2–T7 cyst secondary to subarachnoid hemorrhage, despite partial cyst resolution on MRI. The second experienced postoperative epidural hematoma requiring revision surgery, followed by wound healing complications. At follow-up, this patient had worsened motor function (M5 to M4), increased pain (NRS 3 to 5), and required walking assistance, although imaging showed complete cyst resolution and normalization of the spinal cord signal.

In the SAW group, 4 patients (66.7%) improved on the mMC: 3 improved from grade II to I, and 1 from grade IV to III. No deterioration was observed in this group.

**Fig. 2:**
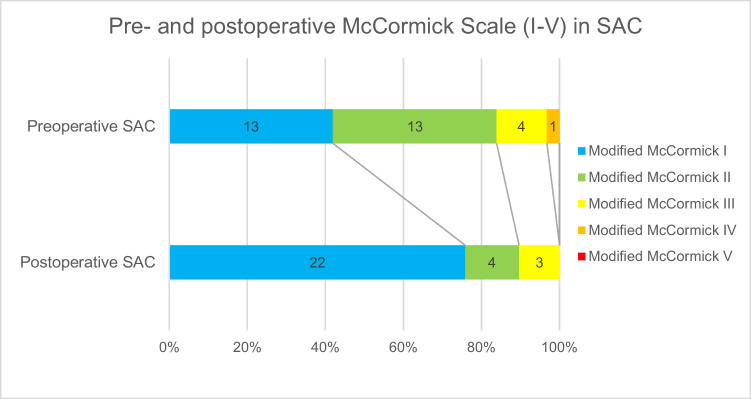
Pre- and postoperative modified McCormick scale in the spinal arachnoid cyst group

**Fig. 3:**
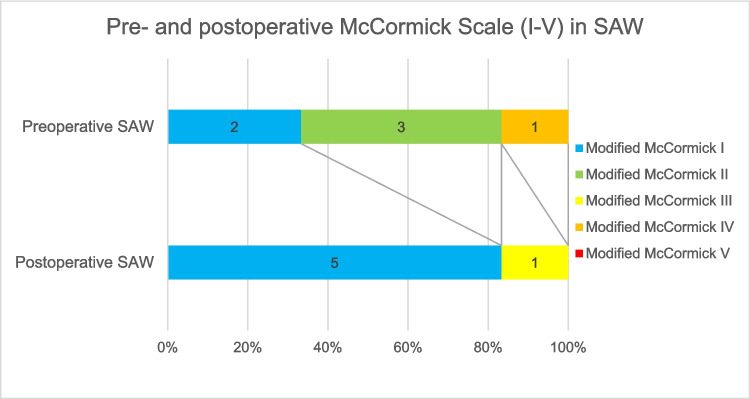
Pre- and postoperative modified McCormick scale in the spinal arachnoid web group

The McNemar test demonstrated no significant improvement in the dichotomized mMC in the SAC (*p* = 0.874) and SAW (*p* = 0.635) group. Favorable outcome (postoperative mMC I-II) was achieved in 26 SAC patients (90%) and 5 SAW patients (83%), with unfavorable outcomes in 3 (10%) and 1 (17%) patient, respectively. Median postoperative mMC at last follow-up was 1 in both groups (SAC IQR 0.0; SAW IQR 0.25) (Figs. 2 and 3).

### Recurrence rate and follow-up

One (2.7%) patient with a SAC had a recurrence after 7.2 months. After revision surgery, symptoms resolved and there was no further recurrence. In the SAW group, there was no recurrence in the FU period.

## Discussion

This study highlights the surgical outcome for SAC and SAW. Although we observed no significant improvement in pre- and postoperative dichotomized mMC, we still observed improvement by at least one grade on the mMC in 37.9% of patients in the SAC, and 66.7% of patients in the SAW group. Our findings demonstrate an overall favorable outcome, with a favorable outcome (mMC I-II) in 90% of patients in the SAC and 83% of patients in the SAW group.

In our series, 60% of SAC were fenestrated and 40% were resected. We found similar results for both techniques, consistent with findings from earlier case series [10]. For SAC, several surgical techniques are discussed in literature. While shunting is usually chosen as a rescue treatment in secondary SAC [21], it is mostly discussed whether complete cyst resection is necessary, or if fenestration is sufficient. Some authors suggest that fenestration is sufficient, since SAC do not contain any secreting cells [3, 11]. It is also often preferred due to the lower invasiveness, especially for cysts spanning over multiple levels [18]. Some authors suggest endoscopic fenestration as an even less invasive treatment [9]. To date the optimal surgical technique for SAC remains debatable, and is mainly dictated by the cyst’s architecture and location, as well as the surgeon’s preference [3].

Surgical treatment is advised in symptomatic patients, with a generally favorable outcome [3]. Reported symptomatic improvement ranges from 66%−92% for SAC [5, 13]. In our series we’ve observed a lower improvement rate of 37.9%. This may be due to the overall relatively good preoperative status of our patients and varying outcome measures throughout different series. It is well recognized, that secondary SAC have worse clinical and radiological outcome with higher rates of radiographic progression (7.8–14.9% vs 21%) and repeat surgery (6% vs 16%) with reports as high as double for secondary SAC [5, 11, 17, 21]. However, we could not reproduce these findings. We observed an overall recurrence rate of 2.7% (1 patient), which occurred in a primary SAC. This may reflect selection bias, as we excluded patients with diffuse, multi-level circumferential SAC.

SAW typically present with myelopathic symptoms [6]. Laminectomy and resection of the web is the predominantly reported surgical technique [1, 6, 19], coherent with our surgical approach. A larger case series from 2023 by Elkadi et. al. [8] of 85 patients with posterior thoracic SAW reports postsurgical improvement rates of 66.7–76.9%. These findings are consistent with ours of 66.7% improvement. Delgardo et al. [6] overserved a lower improvement rate of 58.8% and they observed a worse outcome for patients with an associated syrinx. We observed no recurrence in the SAW group. This is reflected in literature where no recurrences have been observed either [1, 6, 19]. The absence of recurrences may be attributed to the small patient numbers in each series and, in our case, additionally to the relatively short follow-up period. Long-term data on recurrence rates for SAW remain lacking.

In our series, we found a complication rate of 14.7%. Other groups reported higher complication rates of 21% [7]. Permanent deficits are reported in 3% of primary SAC [11]. Reported complication rates for SAW range from 0 to 5.8% [1, 6, 19]. We observed no complications in the SAW group, possibly due to the small sample size, but also possibly because the procedure is more straightforward, typically involving only one level and requiring less spinal cord manipulation.

## Limitations

Firstly, the retrospective design limits the quality of data and statement of strength of causality. Secondly, the small cohort size and the fact that the study was conducted at a single center may affect the generalizability of the findings. Thirdly, the absence of a comparison group of untreated patients prevents any conclusions regarding the natural course of the condition or the potential outcomes for individuals who may not benefit from surgical intervention. Lastly, the short follow-up period, averaging only 3.6 monthly, does not allow for an assessment of long-term outcomes.

## Conclusions

SAC and SAW present most commonly with local or radiating pain and myelopathy. Although neurological status upon presentation is often not severe, symptomatic patients may still benefit from surgery. Consequently, surgery should be considered in symptomatic patients. Surgery for primary SAC and SAW appears to have a generally favorable outcome, though analysis of lager cohorts is needed.

## Data Availability

No datasets were generated or analysed during the current study.

## Abbreviations

BMRC: British Medical Research Council scale
CSF: Cerebrospinal fluid
CT: Computed tomography
IQR: Interquartile range
mMC: Modified McCormick Scale
MRI: Magnetic resonance imaging
NRS: Numeric Pain Rating Score
SAC: Spinal arachnoid cyst
SAW: Spinal arachnoid web
SD: Standard deviation
